# Risks of smoking and benefits of smoking cessation on hospitalisations for cardiovascular events and respiratory infection in patients with rheumatoid arthritis: a retrospective cohort study using the Clinical Practice Research Datalink

**DOI:** 10.1136/rmdopen-2017-000506

**Published:** 2017-09-26

**Authors:** Rebecca M Joseph, Mohammad Movahedi, William G Dixon, Deborah PM Symmons

**Affiliations:** 1 NIHR Manchester Musculoskeletal Biomedical Research Unit, Central Manchester University Hospitals NHS Foundation Trust, Manchester Academic Health Science Centre, Manchester, UK; 2 Arthritis Research UK Centre for Epidemiology, Centre for Musculoskeletal Research, Division of Musculoskeletal and Dermatological Sciences, School of Biological Sciences, Faculty of Biology, Medicine and Health, University of Manchester, Manchester Academic Health Science Centre, Manchester, UK; 3 Department of Rheumatology, Salford Royal NHS Foundation Trust, Salford, UK; 4 NIHR Manchester Biomedical Research Centre, Central Manchester University Hospitals NHS Foundation Trust, Manchester Academic Health Science Centre, Manchester, UK

**Keywords:** smoking, smoking cessation, rheumatoid arthritis, cardiovascular events, respiratory infection, observational study, electronic health records

## Abstract

**Objectives:**

To investigate the associations between smoking status, smoking cessation and hospitalisations for cardiovascular events (CVE) and respiratory tract infections (RTI) in an inception cohort of patients with rheumatoid arthritis (RA).

**Methods:**

The study was set within UK primary care electronic health records (the Clinical Practice Research Datalink) linked to hospital inpatient data (Hospital Episode Statistics). Patients with RA were followed from diagnosis to hospitalisation with a record of CVE or RTI, leaving their general practice, death, or 10 January 2012, whichever was earliest. Smoking status (never, current, former) was defined using primary care data and could vary over time. Survival analysis was performed using Cox regression (first event) and conditional risk set models (multiple RTIs).

**Results:**

5677 patients were included in the cohort: 68% female, median age 61 years. The age-adjusted and sex-adjusted risks of hospitalisation for CVE or RTI were more than twice as high in current vs never smokers (CVE HR (95% CI) 2.19 (1.44 to 3.31); RTI 2.18 (1.71 to 2.78)). The risks for both outcomes were significantly higher in current compared with former smokers (CVE 1.51 (1.04 to 2.19), RTI 1.29 (1.04 to 1.61)). For each additional year of smoking cessation, the risk of first CVE and RTI hospitalisation fell significantly, approximately 25% and 15% respectively in the adjusted models.

**Conclusions:**

Patients with RA who smoke have an increased risk of hospitalisation with CVE or RTI compared with never and former smokers. The risk decreases for each additional year of smoking cessation. Patients with RA who smoke should be advised to stop smoking.

**Key messages**

**What is already known about this subject?**

- Patients with rheumatoid arthritis (RA) are more likely to smoke and to experience cardiovascular events and respiratory tract infections than the general population.

**What does this study add?**

- Patients with rheumatoid arthritis who smoke have double the risk of a hospitalised cardiovascular event compared with patients with RA who have never smoked.
- The risk of a hospitalised respiratory infection is also increased for current smokers.
- In former smokers, the risk of both outcomes falls for each additional year of smoking cessation.

**How might this impact on clinical practice?**

- These results could be used to help encourage patients with RA to stop smoking.

## Introduction

Cigarette smoking is associated with an increased risk of developing rheumatoid arthritis (RA) and therefore there is a higher prevalence of smoking in RA populations.^1^ In the general population, cigarette smoking is known to increase the risk of cardiovascular disease (CVD) and respiratory tract infection (RTI).^2 3^ CVD is more prevalent in patients with RA than the general population,^4 5^ and RTIs also contribute significant morbidity.^4 6^ Cigarette smoking may contribute to the excess risk of CVD and RTI seen in RA populations.

A meta-analysis of cohort studies including members of the general population aged over 60 found the risk of acute coronary events (ACE) was almost doubled in smokers compared with non-smokers (HR 1.98, 95% CI 1.8 to 2.3), while the risk of stroke was increased by almost 60% (HR 1.56, 95% CI 1.4 to 1.8).^2^ RA is associated with an approximate 50% increased risk of incident cardiovascular disease (CVD), including increased risk of myocardial infarction, stroke and congestive heart failure.^7^ Similarly, both cigarette smoking and RA have been associated with an increased risk of RTI. Both current and former smoking have been shown to be associated with community-acquired pneumonia in the general population.^3^ In one study, patients with RA had approximately 90% greater risk of objectively confirmed RTI compared with matched controls (HR 1.88, 95% CI 1.4 to 2.5).^6^

In the general population, there is evidence that smoking cessation is associated with a reduced risk of CVD: in the meta-analysis described above, the risk of ACE and stroke fell by 17% and 13% respectively for each decade of cessation.^2^ The risk of RTI also appears to decrease with smoking cessation, although the benefit was only apparent after 5^3^ or 10^8^ years of cessation and only in patients without chronic obstructive pulmonary disease (COPD).^8^ In a previous study, we found former smokers had a lower risk of cardiovascular mortality than current smokers, and the risk of death due to RTI fell for each additional year of smoking cessation in former heavy smokers.^9^ Smoking cessation could, therefore, potentially reduce the risk of developing CVD and RTI in patients with RA; however, this has not been studied previously.

The aims of this study were to establish, in an inception cohort of patients with RA, (1) the proportions of CVE and RTI hospitalisations in patients with RA who smoke which might therefore be attributable to smoking, (2) the magnitude of any association between cigarette smoking and hospitalisations for major cardiovascular events (CVE) in patients without prior CVD, (3) the magnitude of any association between cigarette smoking and hospitalisations for RTI and (4) whether the risk of being hospitalised for CVE or RTI changes following smoking cessation.

## Methods

### Design and setting

This was a retrospective cohort study set within the Clinical Practice Research Datalink (CPRD),^10^ a database of anonymised electronic health records (EHR) from UK primary care. CPRD contains routinely collected demographic, lifestyle and clinical data for almost 13 million patients.^11^ Clinical information is coded using the Read code system. The EHR provided by CPRD were linked to the National Health Service (NHS) Hospital Episodes Statistics (HES) inpatient dataset which covers all admissions to NHS hospitals in England. Within HES, the International Statistical Classification of Diseases and Related Health Problems 10th Revision (ICD-10) coding system^12^ is used to classify reasons for admission and important comorbidities and the OPCS Classification of Interventions and Procedures V.4 (OPCS-4) coding system^13^ is used to classify procedures. To be eligible for linkage with HES, CPRD patients must have a valid NHS number and be registered with an English practice participating in the linkage scheme: 56% of CPRD practices are participating in HES linkage. Linkage is performed on behalf of CPRD by a trusted third party on the basis of NHS number, gender, date of birth and postcode.

### Study population

Our study population has been described previously.^9^ To be included in the present study, individuals had to be: recorded within CPRD, first diagnosed with RA within the study window (see below), aged 16 years or over at RA diagnosis and eligible for linkage with HES. Patients were excluded if they were prevalent cases of RA, were missing smoking status at baseline, or, for the CVE analysis, if they had a CVE in their HES record prior to RA diagnosis. A validated algorithm was used to identify highly probable cases of RA within CPRD.^14^ The date of RA diagnosis was defined as the date of their first RA Read code (code list available on ClinicalCodes.org^15^) or disease-modifying antirheumatic drug (DMARD) prescription. To define incident RA, all patients were required to have at least 3 years of follow-up in CPRD prior to their RA diagnosis date. If the first DMARD prescription preceded the first RA Read code, an RA Read code needed to be recorded within 3 months of that first DMARD prescription. Patients diagnosed outside the study window (see below) were excluded. For the CVE analysis, patients were required to have at least 2 years of follow-up in the linked HES dataset prior to RA diagnosis in order to exclude those with a prior CVE.

### Study window and follow-up

Linked data were available from 1 January 1998 to 10 January 2012. For the CVE analyses, the study window began on 1 January 2000 (to screen for CVE recorded up to 2 years prior to RA diagnosis) and for the RTI analyses, the study window began on 1 January 1998. For both analyses, the end of the study window was 10 January 2012. All eligible patients entered the cohort on their RA diagnosis date (baseline) and were followed until death, leaving their general practice, the occurrence of an outcome event of interest, the last data collection date for their general practice, or 10 January 2012, whichever was earliest.

### Exposure

Smoking status was determined from the CPRD datasets based on Read codes, additional clinical information and prescriptions for smoking cessation therapy. Smoking status was defined as periods of never, former and current smoking and could vary throughout follow-up. Each period extended from the first record of a particular status until the first record of a different status (for further details see online supplementary figure S1). The algorithm used to define smoking status is available to download.^16^

10.1136/rmdopen-2017-000506.supp1 Supplementary file 1

For former smokers, the number of years of cessation was defined. This value was reset to 0 at the start of each new period of former smoking. As patients had variable amounts of follow-up within CPRD prior to RA diagnosis, information recorded more than 3 years prior to RA diagnosis was not used. The maximum length of smoking cessation at baseline was therefore 3 years. To account for a potential interaction between amount smoked and smoking cessation, amount smoked (light/heavy) was also defined for inclusion in the smoking cessation models. Smokers were categorised as heavy smokers if they smoked more than 20 cigarettes or 10 cigars a day.

### Outcomes

The outcomes of interest were hospitalisation for an atherosclerotic CVE (myocardial infarction, stroke, unstable angina, revascularisation surgery) and hospitalisation for RTI. Hospitalisation was defined as a relevant ICD-10 (CVE and RTI) or OPCS-4 (CVE only) code (online supplementary table S1) recorded at any time during an inpatient spell. We examined the time to first admission for both outcomes. For RTI we also examined the risk of recurrent events, counting each hospitalisation once only.

10.1136/rmdopen-2017-000506.supp2 Supplementary file 2

### Covariates

Gender, socioeconomic status (SES), body mass index (BMI) and year of diagnosis were included as time-independent covariates. The quintile of Townsend score,^17^ at patient postal code level, was used as the indicator of SES. BMI was calculated using each patient’s median recorded height and a weight measurement recorded up to 2 years prior or 1 year post-RA diagnosis (see Joseph *et al* 9 for more details). Year of diagnosis was a marker for calendar year effects. This was categorised as before/after 1 January 2000 to correspond approximately with the introduction of biological therapies for RA in the UK. For the CVE analysis, all person time was in the biologic era and thus calendar year was not considered.

Age was a time-varying covariate. Asthma, COPD, type 2 diabetes and use of immunosuppressant DMARDs were also included as time-varying covariates allowed to switch from absent to present, but not back. Asthma and COPD, included only in the RTI analysis, were defined using code lists created by Doran *et al*.^18^ Read codes used to define type 2 diabetes are available on ClinicalCodes.org.^15^ Use of immunosuppressant DMARDs (methotrexate, azathioprine, ciclosporin, cyclophosphamide, leflunomide and mycophenolate) was included as an indicator of disease severity.

The following medications were time-varying covariates, and switched between ‘on’ and ‘off’ according to the prescription date and a calculated stop date: oral glucocorticoids (GCs), non-steroidal anti-inflammatory drugs (NSAIDs), cardiovascular medication, aspirin/antiplatelet drugs and lipid regulators. The latter three variables were included in the CVE analysis only. For oral GCs, the stop date was calculated using an algorithm which takes into account any recorded dosage and duration information.^19^ The remaining medications were assigned the arbitrary length of 30 days per prescription. Product code lists were defined for all medications based on British National Formulary chapter^20^ and clinical knowledge.

### Analysis

Baseline characteristics according to clinical outcome during follow-up were summarised using proportions or median and IQR. Differences between groups were examined using Χ^2^ test or Kruskal-Wallis test. A significance level of 0.05 was used throughout.

Crude incidence (first CVE and first RTI) and episode (multiple RTIs) rates per 1000 person-years were calculated for each smoking status. The incidence rates were then standardised, using direct standardisation, to match the age/gender structure of the whole incident cohort. Attributable risks (ie, the risk which can be attributed to smoking status alone) were calculated as the differences in standardised incidence rates between the smoking status categories. For CVE, the case fatality risk (CFR) was calculated as the proportion of patients who died of any cause within 30 days of the first CVE during follow-up, excluding patients with <30 days between the CVE and end of the linkage window.

The Cox proportional hazards regression model was used to test the association between a) smoking status and b) length of smoking cessation and the time to first event for each outcome. Models were adjusted for age and gender, then for all covariates. The proportional hazards assumption was checked for each model by inspecting Schoenfeld residuals. If necessary, interactions between covariates and time were included in the models.

For multiple RTI, survival analysis with multiple failures was performed using the conditional risk set model.^21 22^ This model stratifies each patient’s time from follow-up start according to the number of previous events. The risk of failure is calculated for each stratum including only those patients who are still at risk (eg, a patient with one event will be included in strata 1 (no prior events) and 2 (one prior event) only). The time spent in hospital with an RTI plus a 30-day lag-window after discharge was excluded from the time at risk (online supplementary figure S2). The analysis was limited to the first four RTI events due to low numbers of patients in later strata.

10.1136/rmdopen-2017-000506.supp3 Supplementary file 3

Multiple imputation by chained equations^23^ was performed for missing BMI and amount smoked data using linear and logistic regression, respectively. All variables included in the final models were included in the imputation model. Twenty datasets were imputed and results were combined using Rubin’s rules.^24^

Stata/MP V.12.1 (StataCorp, College Station, Texas, USA) was used for data handling and analysis. The study was approved by the Independent Scientific Advisory Committee (protocol reference 13_159).

## Results

Within CPRD, 40 605 patients with RA were identified using the validated algorithm,^14^ 5904 of whom were registered with a practice participating in linkage and met our criteria for incident RA diagnosed during the study window (online supplementary figure S3). A further 227 patients were excluded because of missing smoking status at baseline, leaving a final cohort of 5677 patients. At baseline, there were 2288 (40%) never, 1935 (34%) former and 1454 (26%) current smokers. Former smokers were on average older (median age 60.9, 64.9 and 58 years for never, former and current smokers, respectively) and more likely to be male (never smokers 21%; former smokers 40%; current smokers 38%). Former smokers had a higher baseline prevalence of CVD and type 2 diabetes. The prevalence of asthma was 12% in never smokers, 14% in former smokers and 7% in current smokers (Χ^2 ^(2)=36, p<0.001). The prevalence of COPD was 1% in never smokers, 7% in former smokers and 6% in current smokers (Χ^2 ^(2)=36, p<0. 001).

10.1136/rmdopen-2017-000506.supp4 Supplementary file 4

### Cardiovascular events

Among the 5677 patients, 374 had fewer than 2 years of follow-up in HES and 224 had a CVE prior to RA diagnosis. Of the remaining 5079 patients, 198 (4%) patients had at least one CVE hospitalisation during follow-up. These patients were more likely to be current smokers at baseline (36% vs 25%) (table 1).

**Table 1:** Baseline characteristics according to hospitalisation for major cardiovascular events

	All patients	Never CVE	CVE	Test statistic
n patients (% total)	5079	4881 (96%)	198 (4%)	–
Baseline smoking status, n (%)				
Never	2048 (40%)	1988 (41%)	60 (30%)	–
Former	1749 (34%)	1682 (34%)	67 (34%)	–
Current	1282 (25%)	1211 (25%)	71 (36%)	Χ^2^ (2)=14.4, p=0.001
% female	68.7	69.3	54.6	Χ^2^ (1)=19.2, p=0.000
Median age (IQR)	61.0 (50.9, 70.9)	60.6 (50.4, 70.6)	70.2 (63.5, 77.2)	KW (1)=101, p=0.0001
Median BMI (IQR)^*^	26.9 (23.8, 30.9)	26.8 (23.8, 30.9)	27.6 (24.8, 30.6)	KW (1)=1, p=0.26
Townsend score quintile†				
1	23.5	23.5	22.7	–
2	24.1	24.3	20.7	–
3	21.5	21.4	22.2	–
4	18.4	18.3	20.2	–
5	12.6	12.5	14.1	Χ^2^ (4)=1.9, p=0.754
Type 2 diabetes	10.7	10.5	15.2	Χ^2^ (1)=4.4, p=0.036
Immunosuppressant DMARDs	10.5	10.7	7.6	Χ^2^ (1)=1.9, p=0.167
Oral glucocorticoids	16.5	16.3	22.2	Χ^2^ (1)=4.9, p=0.027
NSAIDs	47.0	46.7	55.6	Χ^2^ (1)=6.0, p=0.014
CVD medication	25.4	24.6	43.9	Χ^2^ (1)=37.6, p=0.000
Aspirin/antiplatelet drugs	7.0	6.7	15.7	Χ^2^ (1)=23.6, p=0.000
Lipid-lowering agents	10.6	10.2	19.2	Χ^2^ (1)=16.1, p=0.000

Figures are percentages, unless otherwise stated.

*33% missing BMI.

†0.5% missing SES.

Never CVE, no hospitalised CVE during follow-up; CVE, at least one hospitalised CVE during follow-up; CVE, cardiovascular event; n, number; BMI, body mass index; DMARDs, disease-modifying antirheumatic drugs; NSAIDs, non-steroidal anti-inflammatory drugs; CVD, cardiovascular disease; Χ^2,^chi-squared test; KW, Kruskal-Wallis test; SES, socioeconomic status.

During 21 843 person-years (pyr) of follow-up, the crude incidence rate of CVE hospitalisations was 9.1 (95% CI 7.9 to 10.4) per 1000 pyr. After adjusting for age and gender, current smokers had the highest incidence rate at 13.7 (9.1–18.3) per 1000 pyr (table 2). The 30-day CFR in the 194 patients with a CVE hospitalisation at least 30 days before the end of the linkage window was 12% (23 patients): never smokers 6% (3/51); former smokers 11% (11/99) and current smokers 20% (9/44). There was no meaningful difference in CFR according to length of smoking cessation: cessation <3 years 10% (3/29); cessation 3 years or longer 11% (8/70).

**Table 2:** Adjusted incidence rate of hospitalisations for cardiovascular events according to smoking status

Smoking status	Age-adjusted and sex-adjusted incidence rate per 1000 pyr (95% CI)	Attributable risk per 1000 pyr (95% CI)
All	9.4 (8.1 to 10.6)	–
Never	6.3 (4.4 to 8.1)	Ref
Former	11 (8.8 to 13.2)	4.7 (3.5 to 5.9)
Current	13.7 (9.1 to 18.3)	7.4 (6.1 to 8.7)

pyr, person-years; CI, confidence interval; ref, reference.

In the fully adjusted Cox regression models, the risk of CVE hospitalisation was more than doubled in current versus never smokers (HR 2.23 (95% CI 1.46 to 3.40)) (table 3). After adjusting for all covariates, current smokers were at significantly higher risk than former smokers (HR 1.51 (95% CI 1.04 to 2.19)) and former smokers were at significantly higher risk than never smokers (HR 1.47 (95% CI 1.04 to 2.08)).

The risk of CVE hospitalisation associated with being a former smoker decreased approximately 25% for each additional year of cessation (table 3). To meet the proportional hazards assumption, an interaction between length of cessation and follow-up time (before/after 5 years) was included in the model; the results therefore represent only the first 5 years after RA diagnosis.

**Table 3:** Cox regression analysis for time to first hospitalised cardiovascular event after rheumatoid arthritis diagnosis

	Unadjusted, HR (95% CI)	Age-adjusted and sex-adjusted, HR (95% CI)	Fully adjusted*, HR (95% CI)
Smoking status			
Current vs never	1.64 (1.1 to 2.44)	2.19 (1.44 to 3.31)	2.23 (1.46 to 3.40)
Current vs former	0.81 (0.57 to 1.15)	1.35 (0.94 to 1.93)	1.51 (1.04 to 2.19)
Former vs never	2.02 (1.44 to 2.83)	1.62 (1.15 to 2.29)	1.47 (1.04 to 2.08)
Smoking cessation			
Per year since cessation, light smoker	0.80 (0.69 to 0.92)	0.76 (0.66 to 0.88)	0.77 (0.66 to 0.91)
Per year since cessation, heavy smoker	0.78 (0.67 to 0.91)	0.74 (0.63 to 0.86)	0.73 (0.62 to 0.87)
Heavy vs light smoker†	1.31 (0.65 to 2.65)	1.68 (0.82 to 3.45)	1.80 (0.79 to 4.10)
Interaction‡	0.98 (0.84 to 1.13)	0.97 (0.83 to 1.12)	0.95 (0.80 to 1.12)

HR, hazard ratio; CI, confidence interval

*Adjusted for gender, age, Townsend score, use of immunosuppressant disease-modifying antirheumatic drugs, use of oral glucocorticoids, use of non-steroidal anti-inflammatory drugs, type2 diabetes, use of cardiovascular drugs, use of aspirin/antiplatelet drugs, use of lipid regulators and body mass index.

†At the time of cessation.

‡Interaction between years of cessation and amount smoked.

### Respiratory tract infection

Of the 5677 patients included in this analysis, 560 (9.9%) patients had at least one, 135 (2.4%) had at least two, 58 (1.0%) had at least three and 27 (0.5%) had at least four hospitalised RTIs. The maximum number of hospitalised RTI recorded was 14. Those with a hospitalised RTI were more likely to be ever smokers (71% vs 58%) and to have asthma (18% vs 10.9%) or COPD (13.4% vs 3.2%) (table 4).

**Table 4:** Baseline characteristics according to hospitalisation for respiratory tract infection

	All patients	Never RTI	RTI	Test statistic
No. of patients (% total)	5677	5117 (90%)	560 (10%)	–
Baseline smoking status, n (%)				
Never	2288 (40%)	2128 (42%)	160 (29%)	–
Former	1935 (34%)	1720 (34%)	215 (38%)	–
Current	1454 (26%)	1269 (25%)	185 (33%)	Χ^2^ (2)=38, p=0.000
% female	67.8	68.6	61.1	Χ^2^ (1)=13, p=0.000
Median age (IQR)	61.4 (51.2, 71.3)	60.3 (50.2, 70.1)	70.8 (62.6, 77.4)	KW (1)=245, p=0.0001
Median BMI (IQR)*	26.8 (23.8, 30.9)	26.9 (23.8, 30.8)	26.6 (23.7, 31.6)	KW (1)=0, p=0.92
Townsend score quintile†				
1	23.1	23.5	19.8	–
2	24	24.4	20.3	–
3	21.8	21.4	25	–
4	18.3	18	21.4	–
5	12.9	12.8	13.6	Χ^2^ (4)=12.9, p=0.012
Immunosuppressant DMARDs	10	10.4	6.4	Χ^2^ (1)=8.9, p=0.003
Glucocorticoids	16.7	15.6	27	Χ^2^ (1)=46.9, p=0.000
Type 2 diabetes	10.7	10.4	13.6	Χ^2^ (1)=5.2, p=0.022
Asthma	11.6	10.9	18	Χ^2^ (1)=25.2, p=0.000
COPD	4.3	3.2	13.4	Χ^2^ (1)=127.9, p=0.000

Never RTI=no hospitalised RTI during follow-up; RTI=at least one hospitalised RTI during follow-up. Figures are percentages, unless otherwise stated.

*33% missing BMI.

†0.5% missing SES.

RTI, respiratory tract infection; n, number; BMI, body mass index; DMARDs, disease-modifying antirheumatic drugs; COPD, chronic obstructive pulmonary disorder; Χ^2^, chi-squared test; KW, Kruskal-Wallis test; SES, socioeconomic status.

During 25 622 pyr of follow-up, the crude incidence rate (counting only the first event) was 21.9 (95% CI 20.1 to 23.7) per 1000 pyr. The age-adjusted and sex-adjusted incidence rates were similar in former and current smokers, each with an attributable risk of 14 (95% CI 12 to 15) additional events per 1000 pyr compared with never smokers (table 5). In total, there were 836 hospitalised RTI recorded during 26 666 pyr of follow-up, giving a crude episode rate of 31.4 (95% CI 29.3 to 33.5) per 1000 pyr. Former smokers had the highest adjusted episode rate. There were 6.7 (95% CI 4.1 to 9.3) additional RTI admissions per 1000 pyr in former compared with current smokers.

**Table 5:** Adjusted incidence rate of hospitalisations for respiratory tract infection according to smoking status

Smoking status	Age-adjusted and sex-adjusted rate per 1000 pyr (95% CI)	Attributable risk per 1000 pyr (95% CI)
First RTI hospitalisation		
All	22.8 (21 to 24.7)	–
Never	14.2 (11.8 to 16.6)	Ref
Former	27.7 (24.4 to 30.9)	13.5 (11.6 to 15.3)
Current	27.8 (22.3 to 33.2)	13.6 (11.7 to 15.5)
All RTI hospitalisations		
All	32.2 (30 to 34.3)	–
Never	17.3 (14.7 to 20)	Ref
Former	43 (39.1 to 46.8)	25.6 (23.4 to 27.9)
Current	36.3 (30.3 to 42.2)	18.9 (16.8 to 21.1)

Table shows incidence rates for the first RTI hospitalisations during follow-up and episode rates for all RTI hospitalisations during follow-up.

pyr, person-years; ref, reference; CI, confidence interval; RTI, respiratory tract infection; Ref, reference.

Considering only the first RTI hospitalisation, current smoking was associated with a significantly increased risk of hospitalisation compared with never smoking in the fully adjusted Cox regression models (HR 1.78 (95% CI 1.38 to 2.29)) (table 6). Current smoking had a 29% increase in risk compared with former smokers; and former smoking had a 38% increase in risk compared with never smokers.

**Table 6:** Cox regression analysis for time to first hospitalised respiratory tract infection after rheumatoid arthritis diagnosis

	Unadjusted, HR (95% CI)	Age-adjusted and sex-adjusted, HR (95% CI)	Fully adjusted*, HR (95% CI)
Smoking status			
Current vs never	1.62 (1.28 to 2.06)	2.18 (1.71 to 2.78)	1.78 (1.38 to 2.29)
Current vs former	0.79 (0.64 to 0.97)	1.34 (1.09 to 1.67)	1.29 (1.04 to 1.61)
Former vs never	2.06 (1.69 to 2.52)	1.62 (1.32 to 1.99)	1.38 (1.12 to 1.7)
Smoking cessation			
Per year since cessation, light smoker	0.92 (0.84 to 1.01)	0.86 (0.78 to 0.94)	0.84 (0.76 to 0.92)
Per year since cessation, heavy smoker	0.91 (0.82 to 1)	0.83 (0.75 to 0.92)	0.83 (0.75 to 0.92)
Heavy vs light smoker†	1.43 (0.9 to 2.27)	1.95 (1.21 to 3.14)	1.37 (0.82 to 2.26)
Interaction‡	0.98 (0.9 to 1.08)	0.96 (0.88 to 1.06)	0.99 (0.9 to 1.09)

HR, hazard ratio; CI, confidence interval.

*Adjusted for gender, age, Townsend score, year of diagnosis use of immunosuppressant disease-modifying antirheumatic drugs, use of oral glucocorticoids, type 2 diabetes, body mass index, asthma and chronic obstructive pulmonary disease.

†At the time of cessation.

‡Interaction between years of cessation and amount smoked.

Each additional year of smoking cessation was associated with an approximately 15% decreased risk of first hospitalised RTI independent of the amount previously smoked (table 6). As for CVE, the results of the smoking cessation models represent only the first 5 years of follow-up.

Using the conditional risk set model for the analysis of recurrent events, the age-adjusted and sex-adjusted HR for current versus never smokers was 2.18 (95% CI 1.74 to 2.74); for current versus former smokers was 1.20 (95% CI 0.99 to 1.46) and for former versus never smokers was 1.81 (95% CI 1.51 to 2.18).

## Discussion

In this cohort of 5677 patients with RA, current smokers had double the risk both of hospitalisation for major CVE and hospitalisation for RTI compared with never smokers. Compared with former smokers, current smokers had an approximately 50% increased risk of hospitalisation for major CVE and 30% increased risk of hospitalisation for RTI. Within former smokers, the risk of hospitalisation for each of the outcomes decreased for each additional year of cessation.

Compared with never smokers, we found that current smoking was associated with seven additional CVE per 1000 pyr follow-up and a more than doubling of the risk of CVE in the regression models. These results are in agreement with a number of previous studies.^25–27^ For example, a meta-analysis found a risk ratio of 1.50 (95% CI 1.15 to 1.84) for current versus non-smokers.^27^ The difference in magnitude could relate to the definition of smoking exposure and the reference category used. Our results are more similar to another CPRD-based study of patients with psoriasis (HR 2.22 (95% CI 2.07 to 2.38) for current vs never smokers),^28^ which also allowed smoking status to vary through time.

In this study, we investigated both the risk of CVE according to smoking status, and the change in risk with increasing length of smoking cessation. The reference category in this second model was patients who have <1 year of smoking cessation, therefore this result should not be interpreted as a decrease in risk compared with current smoking. Additionally, the change in risk is relative to the previous year’s risk rather than the original risk. Taken together the results suggest that overall, current smokers have a higher risk of CVE than former smokers, and within former smokers the risk of CVE decreases approximately 25% for each additional year of cessation. Cohort studies set within the general population^2 29^ have also demonstrated a reduced risk of CVE following smoking cessation in older populations, although the risk was not significantly lower than current smoking until 5^29^ or 10^2^ years after cessation.

The risk of hospitalised RTI was higher in current than never smokers, with an attributable risk of 13.5 additional events per 1000 pyr. After adjusting for age and gender, the risk of RTI was doubled in current compared with never smokers. The HR was attenuated somewhat after including all covariates in the model. The only previous study found no association between smoking status and RTI hospitalisations in patients with RA.^30^ This previous study compared the proportions of each smoking category for those who did and did not have an RTI admission, finding no significant differences. However, the study only included 36 events and may therefore have lacked power.

The risk of hospitalised RTI was also significantly higher in former smokers than never smokers. In the regression models, former smokers had a lower risk of first RTI than current smokers, although the two groups were more similar when considering multiple RTI. The risk of RTI hospitalisation decreased approximately 15% for each additional year of cessation. Few studies have investigated the association between smoking cessation and RTI in the general population: one study demonstrated a decreased risk of hospitalisation for bacterial pneumonia associated with former smoking, but only for those without COPD who had stopped smoking >10 years previously.^8^

Strengths of our study include the large sample size which enabled us to investigate recurrent RTI hospitalisations. Through linkage with HES, we were able to capture all inpatient spells that occurred within England for our cohort. This is the first study to investigate the association between smoking cessation and risk of CVE or RTI in an RA population. Use of prospectively collected EHRs enabled us, for the first time, to define smoking status longitudinally for each patient and allow the status to vary with time. By defining smoking status in this way, the amount of misclassification in smoking status should be markedly reduced compared with using only baseline information.

There are limitations to the study. As discussed previously,^9^ it is likely that some misclassification is still present in our definition of smoking status. It is difficult to assess the impact of potential misclassification, which could result from errors in patient reporting or GP recording of status, or from the frequency of data capture. Those at greater risk of outcomes could be asked about their smoking behaviour more frequently, thus may have fewer classification errors. A second limitation is that the analysis included only events that led to hospitalisation or that occurred while hospitalised, and events were not captured if the patient did not survive to be admitted. We have previously published on the link between smoking and CV and respiratory mortality in this cohort.^9^ For CVE hospitalisations, we aimed to capture incident CVE only and excluded patients with a prior CVE record in their HES dataset. However, 25% of participants were using cardiovascular medication at baseline suggesting that there may have been some prevalent CVD. Excluding these patients would have limited the generalisability of the analysis. The CPRD is considered broadly representative of the UK population^11^ and it is likely that these results are generalisable to all patients with RA within the UK. However, as discussed previously,^9^ diagnosis of RA was based on an algorithm which has a sensitivity of 84% and specificity of 86%^14^ and so a proportion of patients may have been falsely included or excluded. In addition, as we did not include a comparison group without RA, we cannot comment about RA-specific effects. Finally, there is the risk of unmeasured confounding as certain information about RA is not routinely captured in primary care, including direct measures of RA severity and exposure to biologic therapies. We included use of immunosuppressant DMARDs as a proxy measure of RA severity. While there have been conflicting findings, there is some evidence that smoking is associated with more severe RA.^31^

In conclusion, current smoking was associated with a doubling of the risk of hospitalisation both for major CVEs and RTI after adjusting for age and gender. For both outcomes, the risk associated with being a former smoker was lower than for current smokers and decreased for each additional year of cessation. Promoting smoking cessation among patients with RA could therefore reduce the risk of CVE and RTI for these patients. Materials designed to raise awareness of the link between RA and smoking in patients with RA have recently been developed 32 and could help to promote this message.
