# Supplementary material for: Risks of smoking and benefits of smoking cessation on hospitalisations for cardiovascular events and respiratory infection in patients with rheumatoid arthritis: a retrospective cohort study using the Clinical Practice Research Datalink
Source: RMD Open. 2017 Sep 26;3(2):e000506. doi: 10.1136/rmdopen-2017-000506 (PMC5623338; doi:10.1136/rmdopen-2017-000506)
Supplement: Supplementary file 1 [file rmdopen-2017-000506supp001.pdf]

Supplementary Figure S1: Definition of smoking status

1. Identify smoking information from within the CPRD. Three sources: Read codes (status / amount / advice / referrals etc.), additional clinical information (status / amount / advice) and prescriptions for smoking cessation therapy.

↓

2. Combine information from three sources into records: there may be multiple records per day.

↓

3. Set a status for each record based on the combined information, resolving conflicts based on defined rules (e.g. Not Current but prior therapy, therefore Former).

↓

4. Collapse to give a status for each day, resolving conflicts based on defined rules.

↓

5. Clean longitudinal data: Not Current to Never or Former; Ever to Current (or dropped); defined rules for impossible Never.

↓

6. Collapse to a start/stop matrix. Start date = first instance of a new status, stop date = start of next status or follow-up end.

Summary of steps taken to define smoking status using data from the Clinical Practice Research Datalink.

Figure reproduced from Supplementary Figure 2 of the following paper, available under a CC-BY licence (https://creativecommons.org/licenses/by/4.0/legalcode):

Joseph, R. M., Movahedi, M., Dixon, W. G. and Symmons, D. P. M. (2016), Smoking-Related Mortality in Patients With Early Rheumatoid Arthritis: A Retrospective Cohort Study Using the Clinical Practice Research Datalink. Arthritis Care & Research, 68: 1598–1606. doi:10.1002/acr.22882

Available: http://onlinelibrary.wiley.com/doi/10.1002/acr.22882/full (last accessed 13 Dec 2016)
