# Supplementary material for: Risks of smoking and benefits of smoking cessation on hospitalisations for cardiovascular events and respiratory infection in patients with rheumatoid arthritis: a retrospective cohort study using the Clinical Practice Research Datalink
Source: RMD Open. 2017 Sep 26;3(2):e000506. doi: 10.1136/rmdopen-2017-000506 (PMC5623338; doi:10.1136/rmdopen-2017-000506)
Supplement: Supplementary file 2 [file rmdopen-2017-000506supp002.pdf]

Supplementary Table S1 – Outcome code lists

Type	Outcome	Code	Description
ICD-10	RTI	A15	Respiratory tuberculosis, bacteriologically and histologically confirmed
ICD-10	RTI	A15.0	Tuberculosis of lung, confirmed by sputum microscopy with or without culture
ICD-10	RTI	A15.1	Tuberculosis of lung, confirmed by culture only
ICD-10	RTI	A15.2	Tuberculosis of lung, confirmed histologically
ICD-10	RTI	A15.3	Tuberculosis of lung, confirmed by unspecified means
ICD-10	RTI	A15.4	Tuberculosis of intrathoracic lymph nodes, confirmed bacteriologically and histologically
ICD-10	RTI	A15.5	Tuberculosis of larynx, trachea and bronchus, confirmed bacteriologically and histologically
ICD-10	RTI	A15.6	Tuberculous pleurisy, confirmed bacteriologically and histologically
ICD-10	RTI	A15.7	Primary respiratory tuberculosis, confirmed bacteriologically and histologically
ICD-10	RTI	A15.8	Other respiratory tuberculosis, confirmed bacteriologically and histologically
ICD-10	RTI	A15.9	Respiratory tuberculosis unspecified, confirmed bacteriologically and histologically
ICD-10	RTI	A16	Respiratory tuberculosis, not confirmed bacteriologically or histologically
ICD-10	RTI	A16.0	Tuberculosis of lung, bacteriologically and histologically negative
ICD-10	RTI	A16.1	Tuberculosis of lung, bacteriological and histological examination not done
ICD-10	RTI	A16.2	Tuberculosis of lung, without mention of bacteriological or histological confirmation
ICD-10	RTI	A16.3	Tuberculosis of intrathoracic lymph nodes, without mention of bacteriological or histological confirmation
ICD-10	RTI	A16.4	Tuberculosis of larynx, trachea and bronchus, without mention of bacteriological or histological confirmation
ICD-10	RTI	A16.5	Tuberculous pleurisy, without mention of bacteriological or histological confirmation
ICD-10	RTI	A16.7	Primary respiratory tuberculosis without mention of bacteriological or histological confirmation
ICD-10	RTI	A16.8	Other respiratory tuberculosis, without mention of bacteriological or histological confirmation
ICD-10	RTI	A16.9	Respiratory tuberculosis unspecified, without mention of bacteriological or histological confirmation
ICD-10	RTI	A48.1	Legionnaires disease
ICD-10	RTI	J12	Viral pneumonia, not elsewhere classified
ICD-10	RTI	J12.0	Adenoviral pneumonia
ICD-10	RTI	J12.1	Respiratory syncytial virus pneumonia
ICD-10	RTI	J12.2	Parainfluenza virus pneumonia
ICD-10	RTI	J12.3	Human metapneumovirus pneumonia
ICD-10	RTI	J12.8	Other viral pneumonia
ICD-10	RTI	J12.9	Viral pneumonia, unspecified
ICD-10	RTI	J13	Pneumonia due to Streptococcus pneumoniae
ICD-10	RTI	J14	Pneumonia due to Haemophilus influenzae
ICD-10	RTI	J15	Bacterial pneumonia, not elsewhere classified
ICD-10	RTI	J15.0	Pneumonia due to Klebsiella pneumoniae
ICD-10	RTI	J15.1	Pneumonia due to Pseudomonas
ICD-10	RTI	J15.2	Pneumonia due to staphylococcus
ICD-10	RTI	J15.3	Pneumonia due to streptococcus, group B

Type	Outcome	Code	Description
ICD-10	RTI	J15.4	Pneumonia due to other streptococci
ICD-10	RTI	J15.5	Pneumonia due to Escherichia coli
ICD-10	RTI	J15.6	Pneumonia due to other aerobic Gram-negative bacteria
ICD-10	RTI	J15.7	Pneumonia due to Mycoplasma pneumoniae
ICD-10	RTI	J15.8	Other bacterial pneumonia
ICD-10	RTI	J15.9	Bacterial pneumonia, unspecified
ICD-10	RTI	J16	Pneumonia due to other infectious organisms, not elsewhere classified
ICD-10	RTI	J16.0	Chlamydial pneumonia
ICD-10	RTI	J16.8	Pneumonia due to other specified infectious organisms
ICD-10	RTI	J17	Pneumonia in diseases classified elsewhere
ICD-10	RTI	J17.0	Pneumonia in bacterial diseases classified elsewhere
ICD-10	RTI	J17.1	Pneumonia in viral diseases classified elsewhere
ICD-10	RTI	J17.2	Pneumonia in mycoses
ICD-10	RTI	J17.3	Pneumonia in parasitic diseases
ICD-10	RTI	J17.8	Pneumonia in other diseases classified elsewhere
ICD-10	RTI	J18	Pneumonia, organism unspecified
ICD-10	RTI	J18.0	Bronchopneumonia, unspecified
ICD-10	RTI	J18.1	Lobar pneumonia, unspecified
ICD-10	RTI	J18.2	Hypostatic pneumonia, unspecified
ICD-10	RTI	J18.8	Other pneumonia, organism unspecified
ICD-10	RTI	J18.9	Pneumonia, unspecified
ICD-10	RTI	J20	Acute bronchitis
ICD-10	RTI	J20.0	Acute bronchitis due to Mycoplasma pneumoniae
ICD-10	RTI	J20.1	Acute bronchitis due to Haemophilus influenzae
ICD-10	RTI	J20.2	Acute bronchitis due to streptococcus
ICD-10	RTI	J20.3	Acute bronchitis due to coxsackievirus
ICD-10	RTI	J20.4	Acute bronchitis due to parainfluenza virus
ICD-10	RTI	J20.5	Acute bronchitis due to respiratory syncytial virus
ICD-10	RTI	J20.6	Acute bronchitis due to rhinovirus
ICD-10	RTI	J20.7	Acute bronchitis due to echovirus
ICD-10	RTI	J20.8	Acute bronchitis due to other specified organisms
ICD-10	RTI	J20.9	Acute bronchitis, unspecified
ICD-10	RTI	J21	Acute bronchiolitis
ICD-10	RTI	J21.0	Acute bronchiolitis due to respiratory syncytial virus
ICD-10	RTI	J21.1	Acute bronchiolitis due to human metapneumovirus
ICD-10	RTI	J21.8	Acute bronchiolitis due to other specified organisms
ICD-10	RTI	J21.9	Acute bronchiolitis, unspecified
ICD-10	RTI	J22	Unspecified acute lower respiratory infection
ICD-10	RTI	J40	Bronchitis, not specified as acute or chronic
ICD-10	RTI	J44.0	Chronic obstructive pulmonary disease with acute lower respiratory infection
ICD-10	RTI	J44.1	Chronic obstructive pulmonary disease with acute exacerbation, unspecified
ICD-10	RTI	J85	Abscess of lung and mediastinum
ICD-10	RTI	J85.0	Gangrene and necrosis of lung

Type	Outcome	Code	Description
ICD-10	RTI	J85.1	Abscess of lung with pneumonia
ICD-10	RTI	J85.2	Abscess of lung without pneumonia
ICD-10	RTI	J85.3	Abscess of mediastinum
ICD-10	RTI	J86	Pyothorax
ICD-10	RTI	J86.0	Pyothorax with fistula
ICD-10	RTI	J86.9	Pyothorax without fistula
OPCS-4	CVE	K401	Saphenous vein graft replacement of one coronary artery
OPCS-4	CVE	K402	Saphenous vein graft replacement of two coronary arteries
OPCS-4	CVE	K403	Saphenous vein graft replacement of three coronary arteries
OPCS-4	CVE	K404	Saphenous vein graft replacement of four or more coronary arteries
OPCS-4	CVE	K408	Other specified saphenous vein graft replacement of coronary artery
OPCS-4	CVE	K409	Unspecified saphenous vein graft replacement of coronary artery
OPCS-4	CVE	K411	Autograft replacement of one coronary artery NEC
OPCS-4	CVE	K412	Autograft replacement of two coronary arteries NEC
OPCS-4	CVE	K413	Autograft replacement of three coronary arteries NEC
OPCS-4	CVE	K414	Autograft replacement of four or more coronary arteries NEC
OPCS-4	CVE	K418	Other specified other autograft replacement of coronary artery
OPCS-4	CVE	K419	Unspecified other autograft replacement of coronary artery
OPCS-4	CVE	K421	Allograft replacement of one coronary artery
OPCS-4	CVE	K422	Allograft replacement of two coronary arteries
OPCS-4	CVE	K423	Allograft replacement of three coronary arteries
OPCS-4	CVE	K424	Allograft replacement of four or more coronary arteries
OPCS-4	CVE	K428	Other specified allograft replacement of coronary artery
OPCS-4	CVE	K429	Unspecified allograft replacement of coronary artery
OPCS-4	CVE	K431	Prosthetic replacement of one coronary artery
OPCS-4	CVE	K432	Prosthetic replacement of two coronary arteries
OPCS-4	CVE	K433	Prosthetic replacement of three coronary arteries
OPCS-4	CVE	K434	Prosthetic replacement of four or more coronary arteries
OPCS-4	CVE	K438	Other specified prosthetic replacement of coronary artery
OPCS-4	CVE	K439	Unspecified prosthetic replacement of coronary artery
OPCS-4	CVE	K441	Replacement of coronary arteries using multiple methods
OPCS-4	CVE	K442	Revision of replacement of coronary artery
OPCS-4	CVE	K448	Other specified other replacement of coronary artery
OPCS-4	CVE	K449	Unspecified other replacement of coronary artery
OPCS-4	CVE	K451	Double anastomosis of mammary arteries to coronary arteries
OPCS-4	CVE	K452	Double anastomosis of thoracic arteries to coronary arteries NEC
OPCS-4	CVE	K453	Anastomosis of mammary artery to left anterior descending coronary artery
OPCS-4	CVE	K454	Anastomosis of mammary artery to coronary artery NEC
OPCS-4	CVE	K455	Anastomosis of thoracic artery to coronary artery NEC
OPCS-4	CVE	K456	Revision of connection of thoracic artery to coronary artery
OPCS-4	CVE	K458	Other specified connection of thoracic artery to coronary artery
OPCS-4	CVE	K459	Unspecified connection of thoracic artery to coronary artery
OPCS-4	CVE	K461	Double implantation of mammary arteries into heart

Type	Outcome	Code	Description
OPCS-4	CVE	K462	Double implantation of thoracic arteries into heart NEC
OPCS-4	CVE	K463	Implantation of mammary artery into heart NEC
OPCS-4	CVE	K464	Implantation of thoracic artery into heart NEC
OPCS-4	CVE	K465	Revision of implantation of thoracic artery into heart
OPCS-4	CVE	K468	Other specified other bypass of coronary artery
OPCS-4	CVE	K469	Unspecified other bypass of coronary artery
OPCS-4	CVE	K491	Percutaneous transluminal balloon angioplasty of one coronary artery
OPCS-4	CVE	K492	Percutaneous transluminal balloon angioplasty of multiple coronary arteries
OPCS-4	CVE	K493	Percutaneous transluminal balloon angioplasty of bypass graft of coronary artery
OPCS-4	CVE	K494	Percutaneous transluminal cutting balloon angioplasty of coronary artery
OPCS-4	CVE	K498	Other specified transluminal balloon angioplasty of coronary artery
OPCS-4	CVE	K499	Unspecified transluminal balloon angioplasty of coronary artery
OPCS-4	CVE	K501	Percutaneous transluminal laser coronary angioplasty
OPCS-4	CVE	K502	Percutaneous transluminal coronary thrombolysis using streptokinase
OPCS-4	CVE	K503	Percutaneous transluminal injection of therapeutic substance into coronary artery NEC
OPCS-4	CVE	K504	Percutaneous transluminal atherectomy of coronary artery
OPCS-4	CVE	K508	Other specified other therapeutic transluminal operations on coronary artery
OPCS-4	CVE	K509	Unspecified other therapeutic transluminal operations on coronary artery
OPCS-4	CVE	K511	Percutaneous transluminal angioscopy
OPCS-4	CVE	K512	Intravascular ultrasound of coronary artery
OPCS-4	CVE	K518	Other specified diagnostic transluminal operations on coronary artery
OPCS-4	CVE	K519	Unspecified diagnostic transluminal operations on coronary artery
ICD-10	CVE	I21	Acute myocardial infarction
ICD-10	CVE	I21.0	Acute transmural myocardial infarction of anterior wall
ICD-10	CVE	I21.1	Acute transmural myocardial infarction of inferior wall
ICD-10	CVE	I21.2	Acute transmural myocardial infarction of other sites
ICD-10	CVE	I21.3	Acute transmural myocardial infarction of unspecified site
ICD-10	CVE	I21.4	Acute subendocardial myocardial infarction
ICD-10	CVE	I21.9	Acute myocardial infarction, unspecified
ICD-10	CVE	I22	Subsequent myocardial infarction
ICD-10	CVE	I22.0	Subsequent myocardial infarction of anterior wall
ICD-10	CVE	I22.1	Subsequent myocardial infarction of inferior wall
ICD-10	CVE	I22.8	Subsequent myocardial infarction of other sites
ICD-10	CVE	I22.9	Subsequent myocardial infarction of unspecified site
ICD-10	CVE	I23	Certain current complications following acute myocardial infarction
ICD-10	CVE	I23.0	Haemopericardium as current complication following acute myocardial infarction
ICD-10	CVE	I23.1	Atrial septal defect as current complication following acute myocardial infarction
ICD-10	CVE	I23.2	Ventricular septal defect as current complication following acute myocardial infarction
ICD-10	CVE	I23.3	Rupture of cardiac wall without haemopericardium as current complication following acute myocardial infarction
ICD-10	CVE	I23.4	Rupture of chordae tendineae as current complication following acute

Type	Outcome	Code	Description
			myocardial infarction
ICD-10	CVE	I23.5	Rupture of papillary muscle as current complication following acute myocardial infarction
ICD-10	CVE	I23.6	Thrombosis of atrium, auricular appendage, and ventricle as current complications following acute myocardial infarction
ICD-10	CVE	I23.8	Other current complications following acute myocardial infarction
ICD-10	CVE	I63	Cerebral infarction
ICD-10	CVE	I63.0	Cerebral infarction due to thrombosis of precerebral arteries
ICD-10	CVE	I63.1	Cerebral infarction due to embolism of precerebral arteries
ICD-10	CVE	I63.2	Cerebral infarction due to unspecified occlusion or stenosis of precerebral arteries
ICD-10	CVE	I63.3	Cerebral infarction due to thrombosis of cerebral arteries
ICD-10	CVE	I63.4	Cerebral infarction due to embolism of cerebral arteries
ICD-10	CVE	I63.5	Cerebral infarction due to unspecified occlusion or stenosis of cerebral arteries
ICD-10	CVE	I63.6	Cerebral infarction due to cerebral venous thrombosis, nonpyogenic
ICD-10	CVE	I63.8	Other cerebral infarction
ICD-10	CVE	I63.9	Cerebral infarction, unspecified
ICD-10	CVE	I64	Stroke, not specified as haemorrhage or infarction
ICD-10	CVE	I64.0	Stroke, not specified as haemorrhage or infarction
