# Supplementary material for: Risks of smoking and benefits of smoking cessation on hospitalisations for cardiovascular events and respiratory infection in patients with rheumatoid arthritis: a retrospective cohort study using the Clinical Practice Research Datalink
Source: RMD Open. 2017 Sep 26;3(2):e000506. doi: 10.1136/rmdopen-2017-000506 (PMC5623338; doi:10.1136/rmdopen-2017-000506)
Supplement: Supplementary file 3 [file rmdopen-2017-000506supp003.pdf]

Supplementary Figure S2: Infection time at risk

Definition of time at risk when examining recurrent events

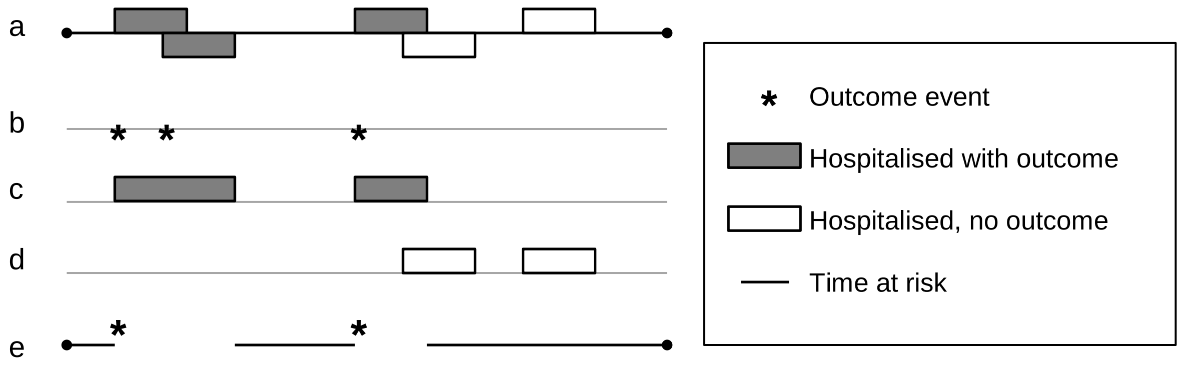

a) Represents a patient's follow-up, showing all hospitalisation spells
b) Timing of outcome events
c) Time spent in hospital with an outcome recorded
d) Time spent in hospital without an outcome recorded
e) Time at risk and outcomes captured. Time at risk excludes time in hospital when an outcome has already been recorded (cannot have more than one outcome per hospitalisation).
