# Supplementary material for: Risks of smoking and benefits of smoking cessation on hospitalisations for cardiovascular events and respiratory infection in patients with rheumatoid arthritis: a retrospective cohort study using the Clinical Practice Research Datalink
Source: RMD Open. 2017 Sep 26;3(2):e000506. doi: 10.1136/rmdopen-2017-000506 (PMC5623338; doi:10.1136/rmdopen-2017-000506)
Supplement: Supplementary file 4 [file rmdopen-2017-000506supp004.pdf]

Supplementary Figure S3: Participant flow through study

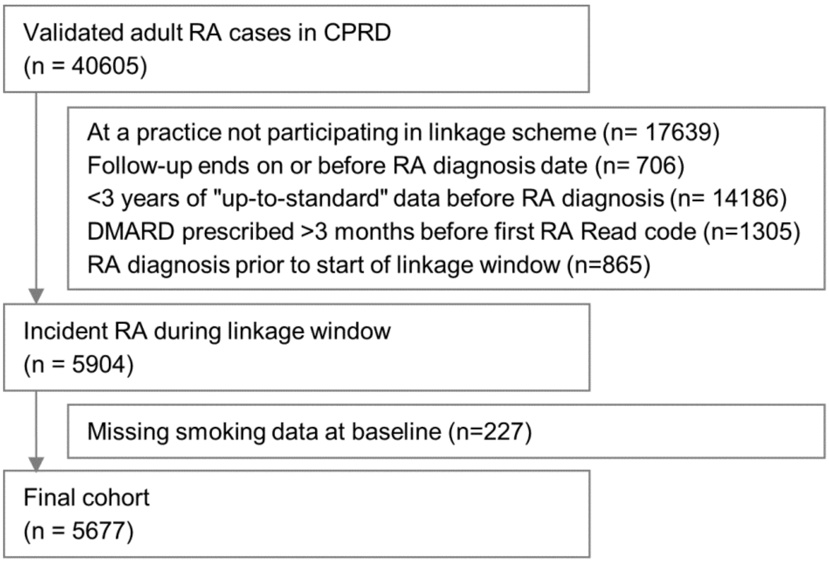

Reduced from the following paper (Figure 1), available under a CC-BY licence (https://creativecommons.org/licenses/by/4.0/legalcode):

Joseph, R. M., Movahedi, M., Dixon, W. G. and Symmons, D. P. M. (2016), Smoking-Related Mortality in Patients With Early Rheumatoid Arthritis: A Retrospective Cohort Study Using the Clinical Practice Research Datalink. Arthritis Care & Research, 68: 1598–1606. doi:10.1002/acr.22882

Available: http://onlinelibrary.wiley.com/doi/10.1002/acr.22882/full (last accessed 13 Dec 2016)
